# Convolutional neuronal networks combined with X-ray phase-contrast imaging for a fast and observer-independent discrimination of cartilage and liver diseases stages

**DOI:** 10.1038/s41598-020-76937-y

**Published:** 2020-11-17

**Authors:** Johannes Stroebel, Annie Horng, Marco Armbruster, Alberto Mittone, Maximilian Reiser, Alberto Bravin, Paola Coan

**Affiliations:** 1 Faculty of Physics, Ludwig Maximilians University, Schellingstr. 4, 80799 München, Germany; 2 Faculty of Medicine, Department of Radiology, Ludwig Maximilians University, Marchioninistraße 15, 81377 München, Germany; 3 European Synchrotron Radiation Facility, 71, Avenue des Martyrs, 38043 Grenoble, France; 4 CELLS: ALBA Synchrotron, Carrer de la Llum, 2-26, 08290 Cerdanyola del Vallès, Barcelona, Spain; 5 RZM—Radiologisches Zentrum München-Pasing, Pippinger Str. 25, 81245 München, Germany

**Keywords:** Medical research, Image processing, Machine learning, Medical imaging, Computed tomography, Optics and photonics, Imaging and sensing, Osteoarthritis

## Abstract

We applied transfer learning using Convolutional Neuronal Networks to high resolution X-ray phase contrast computed tomography datasets and tested the potential of the systems to accurately classify Computed Tomography images of different stages of two diseases, i.e. osteoarthritis and liver fibrosis. The purpose is to identify a time-effective and observer-independent methodology to identify pathological conditions. Propagation-based X-ray phase contrast imaging WAS used with polychromatic X-rays to obtain a 3D visualization of 4 human cartilage plugs and 6 rat liver samples with a voxel size of 0.7 × 0.7 × 0.7 µm^3^ and 2.2 × 2.2 × 2.2 µm^3^, respectively. Images with a size of 224 × 224 pixels are used to train three pre-trained convolutional neuronal networks for data classification, which are the VGG16, the Inception V3, and the Xception networks. We evaluated the performance of the three systems in terms of classification accuracy and studied the effect of the variation of the number of inputs, training images and of iterations. The VGG16 network provides the highest classification accuracy when the training and the validation-test of the network are performed using data from the same samples for both the cartilage (99.8%) and the liver (95.5%) datasets. The Inception V3 and Xception networks achieve an accuracy of 84.7% (43.1%) and of 72.6% (53.7%), respectively, for the cartilage (liver) images. By using data from different samples for the training and validation-test processes, the Xception network provided the highest test accuracy for the cartilage dataset (75.7%), while for the liver dataset the VGG16 network gave the best results (75.4%). By using convolutional neuronal networks we show that it is possible to classify large datasets of biomedical images in less than 25 min on a 8 CPU processor machine providing a precise, robust, fast and observer-independent method for the discrimination/classification of different stages of osteoarthritis and liver diseases.

## Introduction

The analysis and classification of radiological images are highly time-consuming and require trained observers. In the X-ray domain, biological tissues and their pathology-induced modifications can have very similar attenuation properties, thus their discrimination can be very difficult or, in some cases, impossible. Early stages of a disease, characterized by tiny signs and structures, have to be visualized when a new treatment is being studied; this requires the availability of highly sensitive imaging methods and high spatial resolution images. The standard reference in clinical pathology is the histological examination that provides 2D analysis of thin planar slices of small portions of tissue. The method requires labor-intensive protocols (including tissue preparation, cutting, staining/labeling and analysis) necessitating up to tens of hours in case of 3D histology reconstruction^1^. Full organ volumetric representation is still challenging: (1) the minimal sampling in the third dimension is limited by the slice thickness; and (2) the frequent tearing of the tissue occurring during the sectioning phase causes artifacts and thus makes the slice-to-slice alignment prone to error. In addition, histological techniques come short of a complete characterization of the tissue because the to-be-derived information heavily depends on the quality of the staining and labelling of the tissue. Last, the diagnosis relies on skilled operators in all the different steps of the histological exam. X-ray phase contrast imaging (PCI) has proven to provide enhanced sensitivity and accuracy for pathology detection in a not destructive way^2^. The technique is based on the detection of both the amplitude (i.e. attenuation) and the phase changes induced by an object in the X-ray beam. The image contrast produced by PCI can be up to orders of magnitude higher with respect to that given by standard absorption-based radiography in the energy of interest for biomedical imaging and, in particular, for soft tissues^3–5^. As a result, the visibility of low-absorbing structures and of features with similar attenuation properties is largely enhanced. Combined with computed tomography (CT) methodologies, it can provide a highly contrasted 3D representation of the imaged volumes, which can then be virtually sliced along any plane of choice (and at different sampling steps) performing what it is called today in the literature X-ray “virtual histology”^6,7^. Previous works have shown that PCI enables the depiction of different stages of articular cartilage degradation (i.e. osteoarthritis—OA)^8–14^ and of liver fibrosis in a rat animal model^15,16^. In those studies, images were evaluated and classified by experienced radiologists. Osteoarthritis is a degenerative disease, where the cartilage wears up over time^17^ leading to mobility restraints and pain^18^. Liver fibrosis is the excessive accumulation of extracellular matrix proteins including collagen that occurs in most types of chronic liver diseases^19^. Conventional X-ray imaging techniques are sensitive only to advanced stages of these pathologies when therapeutic strategies are less effective.

Convolutional Neuronal Networks (CNN) are new artificial neuronal systems, which offer a highly accurate and observer-independent classification of images^20^.

As reported in the literature, CNNs have been successfully used in different fields, in object detection^21^ and face recognition^22^, and in medical imaging^23^. CNNs were successfully applied, for the first time, to PCI cartilage images by Abidin et al.^24^. This pilot study has inspired us to test multiple advanced CNNs to different computed tomography datasets: one containing cartilage images acquired at much higher resolution than reported in Abidin’s paper using an alternative PCI technique, and a second one including PCI images of liver fibrosis and fat liver.

Our objective is to apply and compare the performance of different CNN systems in terms of their capability in discriminating different stages of cartilage and liver diseases using, as input, datasets images acquired by highly sensitive PCI methods. We aim at identifying the optimal approach and settings in order to establish a procedure for OA and liver data classification that is time-effective, observer-independent and more accurate than what already reported in the literature.

## Methods

### Artificial neural networks

Artificial Neuronal Networks (ANNs) are computing systems inspired by the structure and functioning of biological neuronal networks, which “learn” how to perform tasks from given input examples, without being specifically programmed for the task. ANNs are a sub-category of the more general machine learning algorithms^25^. In this work, the convolutional neuronal network (CNN) was used, which is a special kind of ANN having four different types of layers: an input, a convolutional, a pooling, and a fully connected layer.

The input layer takes the image that is given to the network to be analyzed. The convolutional layer has a kernel with trainable weights and with a size that can be varied: usual sizes are 3 × 3, 5 × 5 or 7 × 7 pixels. The input image is convolved with the kernel, which acts, thus, as a filter. The pooling layer performs downsampling of the input, with parameters that depend on the kernel size and stride length (step of displacement after convolution).

In this study, the so-called max-pooling layer is used: it takes the maximum value within the kernel as an input for the next layer. At the end of the network system, classification and activation functions are performed in the fully connected layer, where all artificial neurons are connected^26^.

In the CNN language, one epoch is one iteration of the network. An epoch consists of two parts: the forward processing of images to classify them and the backpropagation to change/train the weights and converge towards an improved classification. In the forward processing, the image data go from the input layer to the classification layer; in this latter case, a function calculates the error between the predicted classification and the apriori classification information (error function) by considering the effect of every weight. To minimize the error, an optimizer^27^ is used, which adjusts the weight according to the learning rate set by the user (in the range [0,1]). Depending on the number of epochs and the learning rate, the CNN converges and classifies the data; therefore, the learning rate is set to get the fastest convergence (as defined at the end of this section) and the best classification.

In this study, we used the so-called transfer learning method that works with CNN weights pre-trained on large image datasets^28^. In our case, weights were trained on the ImageNet dataset^29^. A custom-designed network was implemented: this was achieved by removing the classification layer of the pre-trained network and by adding a fully connected layer and another classification layer with two or four outputs, depending on the dataset. The pre-trained CNN acts as a feature extractor for the self-designed part of the network. In the backpropagation, the weights are adjusted in the self-designed network, whereas the weights in the pre-trained network are fixed.

We tested three pre-trained CNNs for our study: the VGG16^30^, the Inception V3^31^, and the Xception Network^32^. We selected these specific networks, because of their high performance in the image classification competition, i.e. the Large Scale Visual Recognition Challenge (LSVRC) organized by the ImageNet project^33^. The achieved accuracy in the challenge was 90.1% for VGG16, 94.1% for the Inception V3 network and 94.5% for the Xception^32^.

The VGG16 CNN is a network with 16 convolutional layers with a kernel size of 3 × 3 pixels, two fully connected layer and a classification layer with 1000 classification outputs. The size of the input images is 224 × 224 pixels (for RGB images)^30^. The VGG16 is a heavy computation network, with long training times and a large number of weights (for a total size of 533 MB).

The Inception network was introduced by Szegedy et al.^34^. The idea is to construct the network “wider” instead of “deeper”. To make a network “deeper”, layers are added in such a way that layers are behind each other. For the Inception network, layers are instead added and arranged in a parallel configuration; the network becomes thus “wider” and the layers also work in parallel. In this way, the size of the pre-trained weights is reduced to 96 MB^35^.

The Xception Networks architecture builds on a depth-wise separable convolutional layer. The weight size of this network is 91 MB^32^.

The analysis was performed on a Fujitsu workstation with 8 Intel Xeon CPU processors with 4 kernels and 2.6 GHz. The graphics card on which the calculations were carried out is a NVIDIA Quadro P1000 with 4 GB Memory. The entire code is written in Python based on the Keras library^36^, a deep learning library in Python interfacing tensorflow-GPU^37^ as a backend.

### Transfer learning process

We used the transfer learning of the CNNs, which is achieved through a three-step process.

1. The network is divided in two sections: the first section is trained first on a large dataset with annotated images, which is not related to the later task.
2. All the parameters in the first section are fixed and a second training is performed using a dataset related to the classification task to train the second section of the network. In this way, the algorithm learns how to classify the images.
3. The algorithm is applied to the dataset of interest in a fully automated manner.

### Fine tuning of the networks

For the fine tuning of the networks, we tried to improve them in two ways: (1) by studying the influence of parameters such as optimizer, learning rate or number of epochs etc.; (2) by adding an additional network element to the pre-trained network, such as fully connected layer, drop-out layer, etc.

Concerning the first method, we kept the optimizer algorithm RMSProb^38^ for all cases constant as well as the number of epochs. The number of epochs was chosen to assure convergence of the CNN. In this study, we defined a convergence criteria based on a threshold (± 0.5% between two consecutive epochs of the validation data). The learning rate has been always adjusted to push the network to its best performance and to avoid overfitting.

Using the second method, the best results were obtained by adding one fully connected layer.

### Phase contrast imaging and dataset description

In X-ray PCI the image contrast derives from the perturbations of the X-ray wave-front induced by the presence of an object along its propagation path. This contrast mechanism has been proven leading to a superior image contrast^3–5^ with respect to standard X-ray attenuation, especially in case of soft tissues. In this work, we applied X-ray PCI to investigate cartilage and liver biological specimens.

### Cartilage samples and dataset

**Figure 1:**
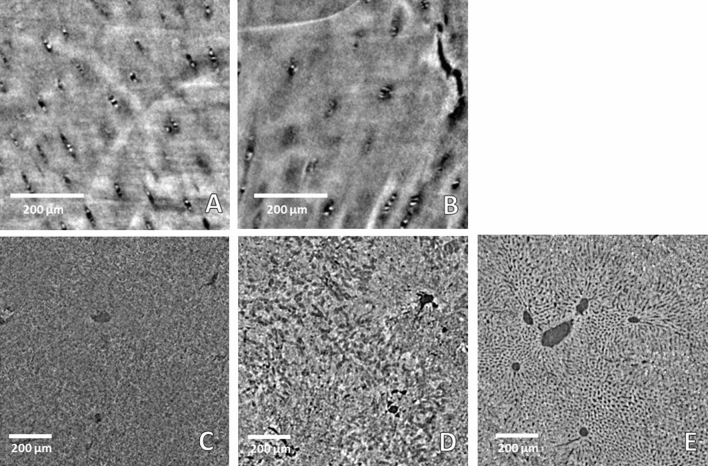
Examples of PCI micro CT images (224 × 224 pixels) used as input of the neuronal network's systems; (**A**,**B**): images acquired with a detector pixel size of 0.7 × 0.7 µm^2^ of a healthy (**A**) and degenerated (**B**) cartilage specimen, respectively; (**C**–**E**): PCI images acquired at a final voxel size of 2.2 × 2.2 × 2.2 µm^3^ of a healthy (**C**)**.** (**D**) fibrotic-4 weeks liver and (**E**) fat liver, respectively.

For the cartilage evaluation, human cadaveric patellae were used. According to the regulations for experiments involving cadaveric samples, this study was waived by the ethics committee of the Ludwig-Maximilians-University, Munich, Germany. However, the required informed consent was obtained from the legally authorized/next of kin of the deceased prior to the extraction of the patella. Samples were extracted in compliance with the relevant guidelines and regulations by the forensic medicine department of the Ludwig-Maximilians-University, including testing for infectious diseases. Four cartilage samples (plugs), cylinders of 7 mm in diameter, were harvested from human patella (67-year-old woman) within 24 h of death. The plugs were divided into two groups based on OARSI assessment system^39^ by two experienced pathologists: the control group with healthy cartilage samples and the OA degraded cartilage group. The samples were imaged at the Biomedical beamline (ID17) of the European Synchrotron (ESRF, France) by using X-ray propagation-based PCI micro-CT^40^ with a polychromatic and filtered X-ray beam with peak energy around 40 keV^41^. The detection system consisted of a PCO edge 5.5 sCMOS camera^42^ coupled with a 10 × optics and a 19 µm thick GGG scintillator screen leading to a final pixel size of 0.7 × 0.7 µm^2^. From the reconstructed CT volumes, sagittal CT images of the transitional and mid zone of the cartilage are extracted layer (1024 × 1024 pixels), downscaled to reduce them to 224 × 224 pixels in order to fit the CNN requirements and finally normalized to values in the [0–1] range. The analysis was performed on images presenting a voxel size of 0.7 × 0.7 × 0.7 µm^3^. Example are shown in Fig. 1A,B): (1A) is a sagittal PCI CT image of a healthy cartilage, whereas is (1B) the sagittal slice of an osteoarthritic cartilage sample with a small crack of the tissue visible on the right side. From every group, 3800 images were extracted and split into three categories: 60% were used as training data, 20% as validation data during training and 20% as testing data after training. Half of the images corresponded to healthy samples, the other half to pathological ones. In addition, for this step we have split the samples as follows: two samples (one from each group) were used for training the network; the images of the remaining two samples were split equally into validation and testing data sets^43,44^.

### Liver samples and dataset

Six Male Lewis rats (Charles River Wiga, Sulzfeld, Germany; 170–190 g of weight) underwent syngeneic orthotopic liver transplantation with a surgical technique described in detail elsewhere^45,46^. Organs were stored in cold University of Wisconsin solution (4 °C; DuPont de Nemours, Bad Homburg, Germany). All experiments were carried out in accordance with the German legislation on animal protection and with the “Principles of Laboratory Animal Care” (NIH publication no. 86-23, revised 1985)^15^. All experimental protocols for the rat studies were approved by the local government (Regierung von Oberbayern, Munich, Germany) and were reported to the responsible authorities regularly. The liver samples were divided into three groups: (1) healthy; (2) fibrotic four weeks (4 weeks perfusion); (3) fatty livers. The samples were all paraffin-embedded and were imaged with X-ray PCI micro-CT with a polychromatic X-ray beam with a mean energy of 24 keV at the ID19 beamline at the ESRF. The detection system was a PCO Edge with a 6.5 µm pixel size connected with a 2.9 × optics, thus determining a final effective pixel size of 2.2 × 2.2 µm^2^. The analysis was performed on CT slices presenting a voxel size of 2.2 × 2.2 × 2.2 µm^3^. Both CT datasets were pre-processed and reconstructed with the PyHST2 software^47^.

The 512 × 512 pixels’ liver images were extracted from 3D reconstructed volumes, the intensity normalized to the [0–1] range and reduced as well to 224 × 224 pixels by binning via linear interpolation. This dataset originates from 6 different liver samples: two healthy (Fig. 1C), two fibrotic 4 weeks (Fig. 1D) and two fatty livers (Fig. 1E). A total of 3600 images were obtained and, from each liver sample, 600 images were extracted. By applying again, a 60/20/20 split ratio, 2160 images were used for training the network, 720 for validation during the training and 720 image were used for testing the trained network. In addition, the total number of input images for training and testing was increased by rotating the original images by 90, 180 and 270 degrees and adding them to the respective groups of images. This method is referred as “data augmentation” and it increased the total number of available images by a factor of four in this case^48^. In this third step, we have split the samples as follows: one of each group was used for training and the images of the remaining three samples were used for validation and testing data sets^43,44^.

The training data were used for training and updating the weights of the network. The validation data were used to evaluate after each iteration the accuracy of the network. The testing data set was used to evaluate the accuracy of a trained model with a new dataset. The accuracy of the performances of the networks was calculated as the ratio of the sum of the true positive cases plus true negative cases over the total number of input images:

$$Accuracy=\frac{\sum True\,Positive+ \sum True\,Negative}{\sum Total\,number\,of\,images}$$

## Results

**Figure 2:**
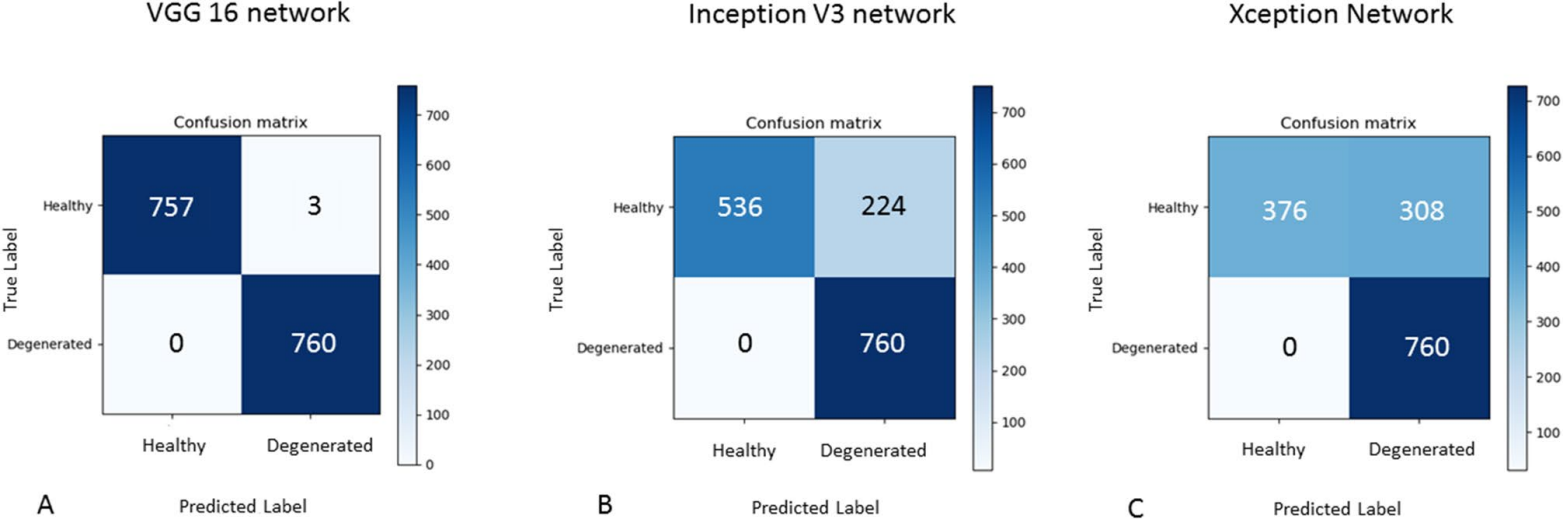
Confusion matrix of the VVG16, Inception V3, and Xception networks applied to the cartilage dataset. They show the “true” label (as a result of the histologic analysis) and the predicted (by the CNN) label. (**A**) The test accuracy in classifying healthy and degenerated (i.e. OA affected tissues) is 99.8% with the VGG 16 (**B**) The test accuracy of the Inception V3 network is 84.7% (**C**) The Xception has a test accuracy of 72.6%. The confusion matrices in this figure were generated with matplotlib version 2.2.2 (https://www.matplotlib.org.cn/en/).

**Figure 3:**
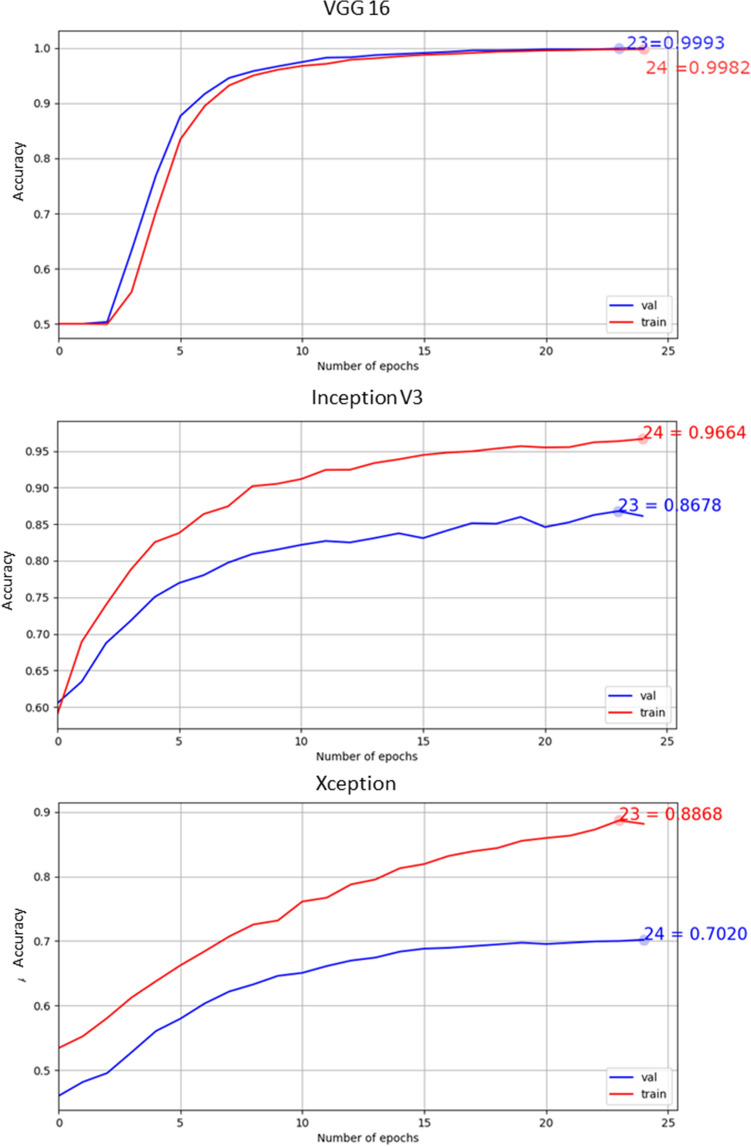
Accuracy plots as a function of the number of epochs for the VGG16, Inception V3 and Xception networks. The Xception has the largest gap between training and validation accuracy and the highest level of accuracy is achieved by the VGG16 network. The line-plots in this figure were generated with matplotlib version 2.2.2 (https://www.matplotlib.org.cn/en/).

All validations were done with respect to the histological data, taken as reference. For the cartilage data, the VGG16 network provided a testing accuracy of 99.8% (validation accuracy 99.8% and training accuracy 99.9%) (Fig. 3 top) after 25 epochs and a learning rate of 7 × 10^–7^. In this case, none of 760 images were falsely classified as healthy instead of degenerated (false-positive) and 3 out 760 images were classified healthy instead of degenerated (false-negative), as shown in the so-called confusion matrix in Fig. 2A. The time to train and validate this network was 34 min and 42 s.

The inception V3 network classified the data with a test accuracy of 84.7% (validation accuracy 86.8% and training accuracy 96.6%) (Fig. 3 mid) after 25 epochs with a learning rate of 1 × 10^–6^; in numbers: 224 images were predicted as false negative and no false positive cases were found (Fig. 2B); 536 healthy images were classified correctly and 760 images with signs of degeneration were classified correctly over the total number of 1520 images (Fig. 2B). The calculation time for this network (training, validation and testing) was 19 min and 41 s. The Xception network has 308 images predicted false positive and no false negative; 376 healthy images and 760 degenerated are predicted correctly (Fig. 2C). Figure 3 shows the accuracy (training and validation) plot as a function of the number of epochs for the VGG16, the Inception and the Xception networks, respectively; it shows how they converge toward a stable solution for a same number of iterations.

With the Xception network, a validation accuracy of 70.2% and a testing accuracy of 72.6% was achieved after 25 epochs. The training, validation and testing with this Network took 37 min and 25 s. The accuracy of the training data is at 88.7% (Fig. 3 bottom). By using instead 42 epochs, the plateau (< ± 0.5% different between epochs) was reached during the training accuracy (97.2%), the validation accuracy increased to 79.7% and the testing accuracy was 81.3%. The time required for this calculation was 1 h 14 min and 33 s. Training the network with more epochs the testing accuracy of the Xception network is increased, but it does not reach the testing accuracy of the inception or VGG16 network.

In the next step, the cartilage data were split by sample type: images of one healthy and one degenerated sample were used for training. The images of the other samples were used for validation and for testing. Therefore, we have a split of 50/25/25 percent: 1900 images were used for training (950 images from healthy sample, 950 image from degenerated sample) and 950 images were used for validation and testing. The training with this dataset was 25 epochs long. The testing (validation) accuracy of the VGG16 network was 68.6% (70.0%) whereas the training accuracy is 99.9%. The training/validation and testing took 32 min and 45 s. The inception network achieved a testing (validation) accuracy of 65.9% (66.6%) and a training accuracy of 99.0%. The calculation time was 29 min and 38 s. The testing (validation) accuracy was at 75.7% (79.8%) of the Xception and a training accuracy of 99.8%. The testing accuracy of the VGG16 network, declined as well as the accuracy of the inception network, when splitting the dataset based on samples. The Xception network increased its testing accuracy from 72.6% up to 75.7%.

**Figure 4:**
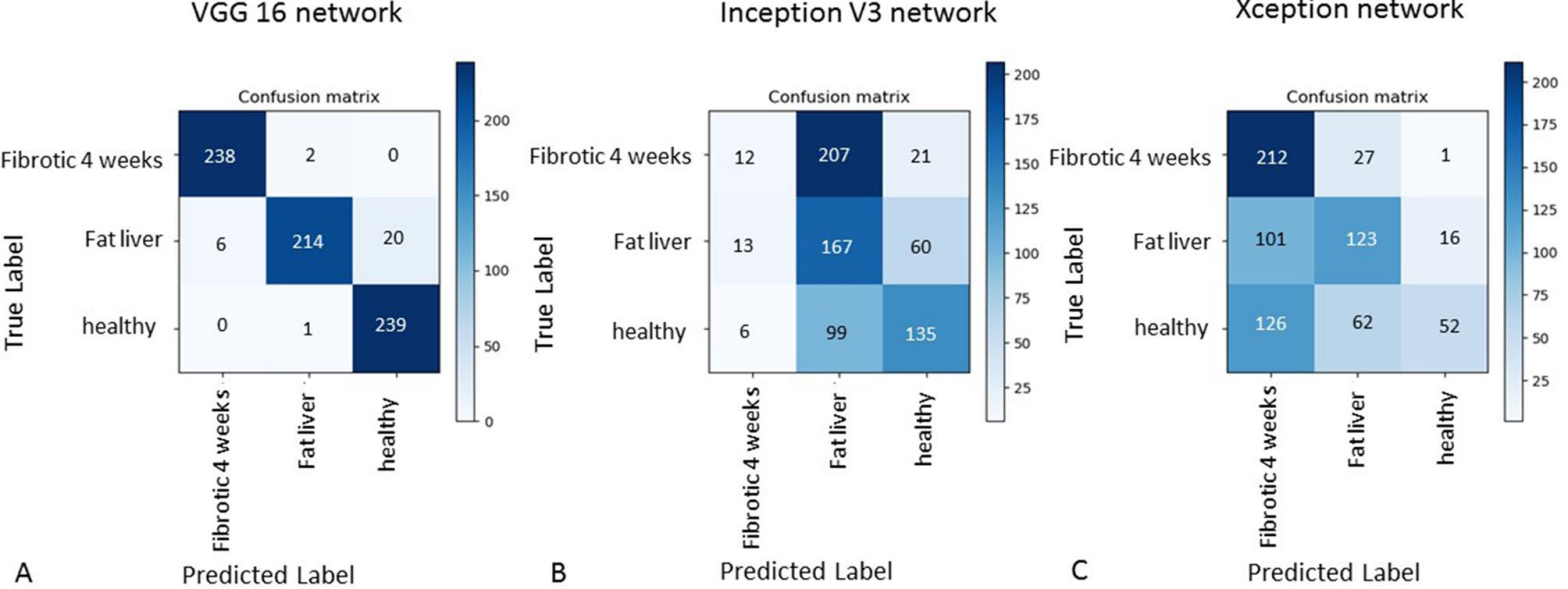
Confusion matrixes representing the image classification capability for the VGG16 (**A**), Inception V3 (**B**) and Xception (**C**) networks. The testing accuracy of VGG16 network is 96.0%, for Inception 43.6% and for Xception 53.8%. The confusion matrices in this figure were generated with matplotlib version 2.2.2 (https://www.matplotlib.org.cn/en/).

For the liver data, we report in Fig. 4 the confusion matrixes. For all the three CNNs, the convergence was obtained after 50 epochs. The VGG16 network performed with a test accuracy of 96.0% (validation accuracy 96.1%), with a learning rate of 1 × 10^–6^. The training accuracy is slightly lower at 94.6%. 2 healthy, 26 fat liver, 1 fibrotic (4 week) images got mistakenly classified (Fig. 4A). The computational time was 34 min and 21 s.

The Inception V3 network, with a learning rate of 1 × 10^–6^, performed on this dataset with a test accuracy of 43.6% in 18 min and 54 s. By this network, 442 out of 720 images were falsely classified (Fig. 4B). The Xception network (learning rate of 1 × 10^–6^) performed with a test accuracy score of 53.8% on this dataset in 36 min and 1 s (Fig. 4C).

To increase the number of samples as input for the network, we decided to repeat the classification by rotating the images to get a more general classifier. The training/validation/testing ratio was set to 60/20/20 again. As a result, 14,400 images (4 × 360 original images) were available: 8640 images were used for training the CNNs and 2880 for the validation; finally, 2800 were used for testing the network. The networks converged faster with a larger number of images, thus the number of epochs was reduced for this calculation down to 15 epochs. With the VVG16 network, a test (validation) accuracy of 95.6% (95.0%) was achieved. The Inception V3 network reached a testing (validation) accuracy of 38.8% (35.8%) and the Xception network a test (validation) accuracy of 50.3% (48.8%), but the training accuracy was at 86.2% and 95.4%. The calculation times increased compared to the previous liver cases: 42 min and 11 s for the VGG16, 23 min and 10 s for the Inception network and 44 min and 19 s for the Xception network.

In a next step, we tested the network performances by training the system with images of one set of samples and then validating and testing it with another set. For training 1800 images from three samples of different groups were used (600 images one healthy liver sample, 600 images from one fatty liver sample and 600 images from one four-week perfusion sample). The classification accuracy of the testing dataset by the VGG 16 network was 75.4%, whereas the training (validation) accuracy score was 99.8% (73.7%). Training (15 epochs) and testing process of this network took 10 min and 33 s. The inception network obtained a testing accuracy of 39.8% and a training (validation) accuracy of 99.94% (41.0%). This training (15 epochs) and testing of the network took 6 min and 10 s. The Xception network achieved a testing accuracy of 42.0%. The training and validation accuracy were 98.7% and 46.4%, respectively. The training of the network with 15 epochs and testing lasted 11 min and 17 s.

## Discussion and conclusions

In this work, we have investigated the possibility of using convolutional neuronal networks for the classification of healthy and pathological biological tissues considering two different biomedical cases: osteoarthritic cartilage and liver fibrosis. The evaluation of the samples was carried out by two experienced pathologists on the basis of the histological results, which served as golden standard.

We have applied three CNNs (VGG16, Inception V3, and Xception networks) and compared their performances in terms of accuracy in the classification and the time needed for this calculation. The VGG16 network provided the highest accuracy, compared to the Inception V3 and Xception network in the analyzed cases. In the VGG16, the entire image is convoluted, whereas in the Inception V3 and the Xception networks, the image to be analyzed is split into different regions. This process of subdividing the images can lead to overfitting that causes poor performances of the networks when applied to data in the validation and testing phase. This fact determines the discrepancy between the training and the validation/testing accuracy curves for the Inception V3 and Xception networks. Additionally, this explains the discrepancy of our results with respect to the LSVRC competition.

We also tested the effect of training the network with images of two cartilage samples (one healthy and one degenerated) and validated and tested with images of another cartilage sample: the testing accuracy decreased in all of the three networks. However, the Xception network was the one with the highest testing accuracy. This last fact shows that the Xception network model is the best generalized model, when splitting the dataset by sample type.

Many different ways, such as additional fully connected layers and drop out layers, changing the optimizer algorithm or adjusting the learning rate, were used to reduce overfitting in the Xception and Inception V3 networks. The results we presented here were obtained after this optimization procedure (best accuracy and lowest overfitting); instead, the results of these intermediate optimization procedures were not reported.

In the case of the cartilage, other computer-aided diagnosis tools are available, like texture analysis. This kind of analysis on cartilage PCI images for characterizing osteoarthritis, gives good results for both 2D images^49^ and 3D volumes^50^.

The Inception V3 network with its inception modules is much faster for training, validation and testing than the other two networks. For the cartilage dataset, the Inception was 56.7% faster than the VGG16 network and 47.4% than the Xception. For the liver dataset, the Inception was 26.6% and 29.5% faster than the VGG16 and Xception networks, respectively. The reason of its higher performances lies in its unique inception module structure, which reduces the number of trainable weights and therefore speeds up the computation.

With the data augmentation of the liver dataset, we could show that the networks converged faster when the number of input images was increased and therefore we needed fewer epochs. For the VGG16 network, the testing accuracy stayed approximately the same 95.5% and but the computational time increased by 23.5% from 34 min and 21 s to 42 min and 11 s because of increasing the number of input images by a factor of 4. We can conclude that more input data leads to a better accuracy and a faster convergence of the VGG network, but this does not come with shorter computational times.

When we used data from different liver samples for the training and the testing of the networks, we achieved a decreased testing accuracy of all the networks, whereas the training accuracy increased. This result shows that an overfitting occurs and the networks do not generalize enough; to overcome this limitation, a larger number of samples should be used. The network presenting the best testing accuracy is the VGG16 with 75.38%, as in the calculation without the split based on samples. Both the Inception V3 and Xception networks testing accuracies were for this test below 50%; for this test and both networks did not perform well on liver data, in contrast to the cartilage data, were both networks had a testing accuracy above 68%.

The testing accuracy strongly depends on the data splitting method that is used. If the slices for training and testing the CNN are extracted from the same sample, the data used in the two processes may look very similar and an overfitting of the networks during training may occur. In this case, the generalization of the CNN on new samples is unsettled and may be severely hindered.

This study shows that the combination of advanced high sensitive X-ray imaging techniques (PCI) with newly available algorithms for data classification based on the neuronal network concept, could significantly support the discrimination between healthy-normal and pathological-abnormal conditions of biological tissues. The proof of concept of this methodology was here performed on small tissue samples (cylindrical bone/cartilage plugs of 7 mm in diameter). This method could be an important asset in the direction of the automation of diagnostic procedures. The application of CNNs to our datasets showed that these tools (in the specific case we identified the VGG16 network as the most accurate one) make it possible to analyze and classify sets of 9616 images of 224 × 224 pixels in less than 25 min providing a robust, fast and observer-independent method of diagnosis.

## Data Availability

The data used in this study are available from the corresponding author, upon reasonable request.
